# Supplementary material for: Mass cytometry reveals systemic and local immune signatures that distinguish inflammatory bowel diseases
Source: Nat Commun. 2019 Jun 19;10:2686. doi: 10.1038/s41467-019-10387-7 (PMC6584653; doi:10.1038/s41467-019-10387-7)
Supplement: Supplementary file 1 — Supplementary Information [file 41467_2019_10387_MOESM1_ESM.pdf]

Supplementary Information for:

Mass cytometry reveals systemic and local immune signatures that distinguish inflammatory bowel diseases

By Rubin *et al.*

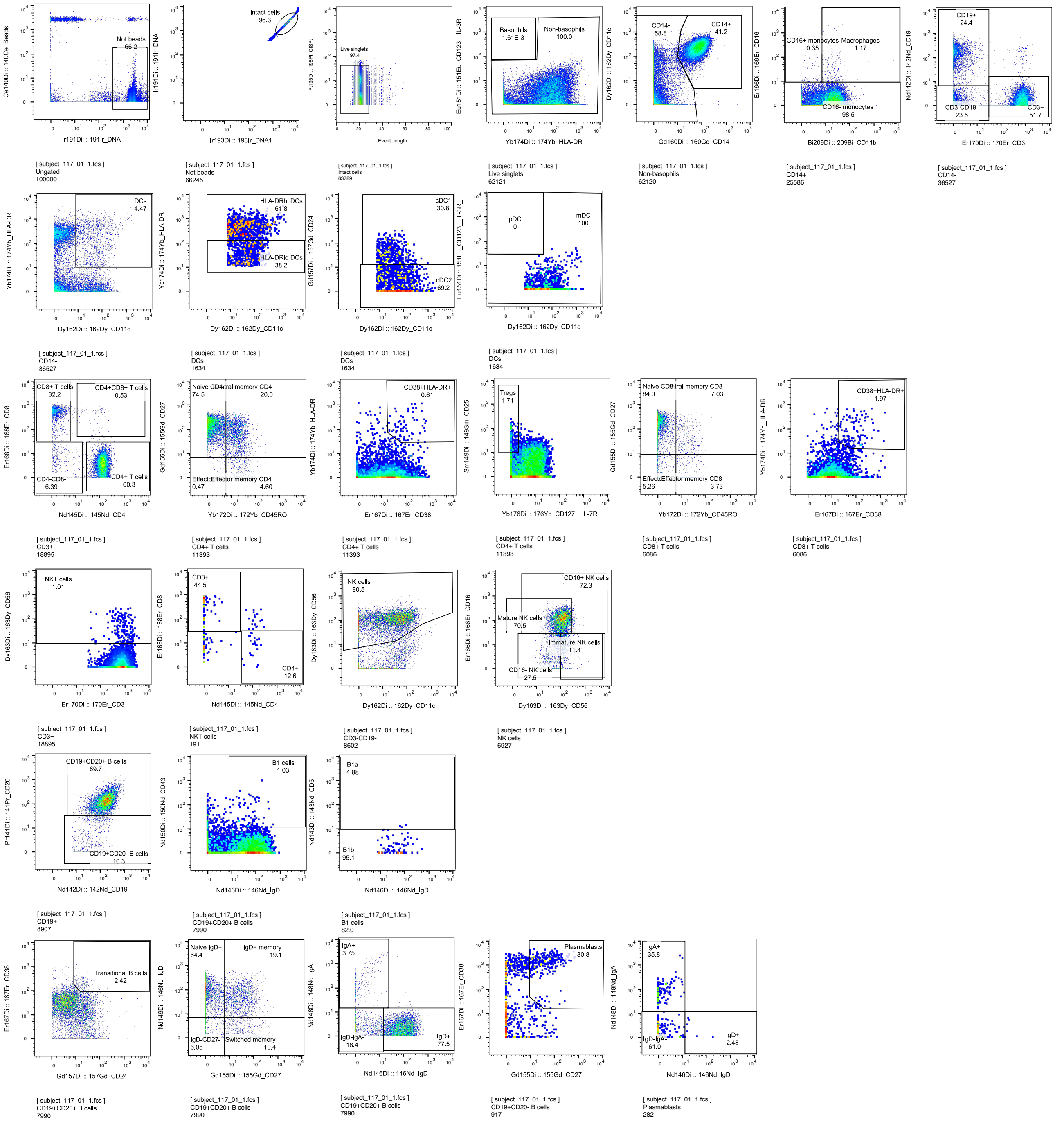

Supplemental Figure 1. Representative core gating scheme utilized for cohort 1 blood samples. Further subpopulations defined by positive or negative expression of additional markers are not shown.

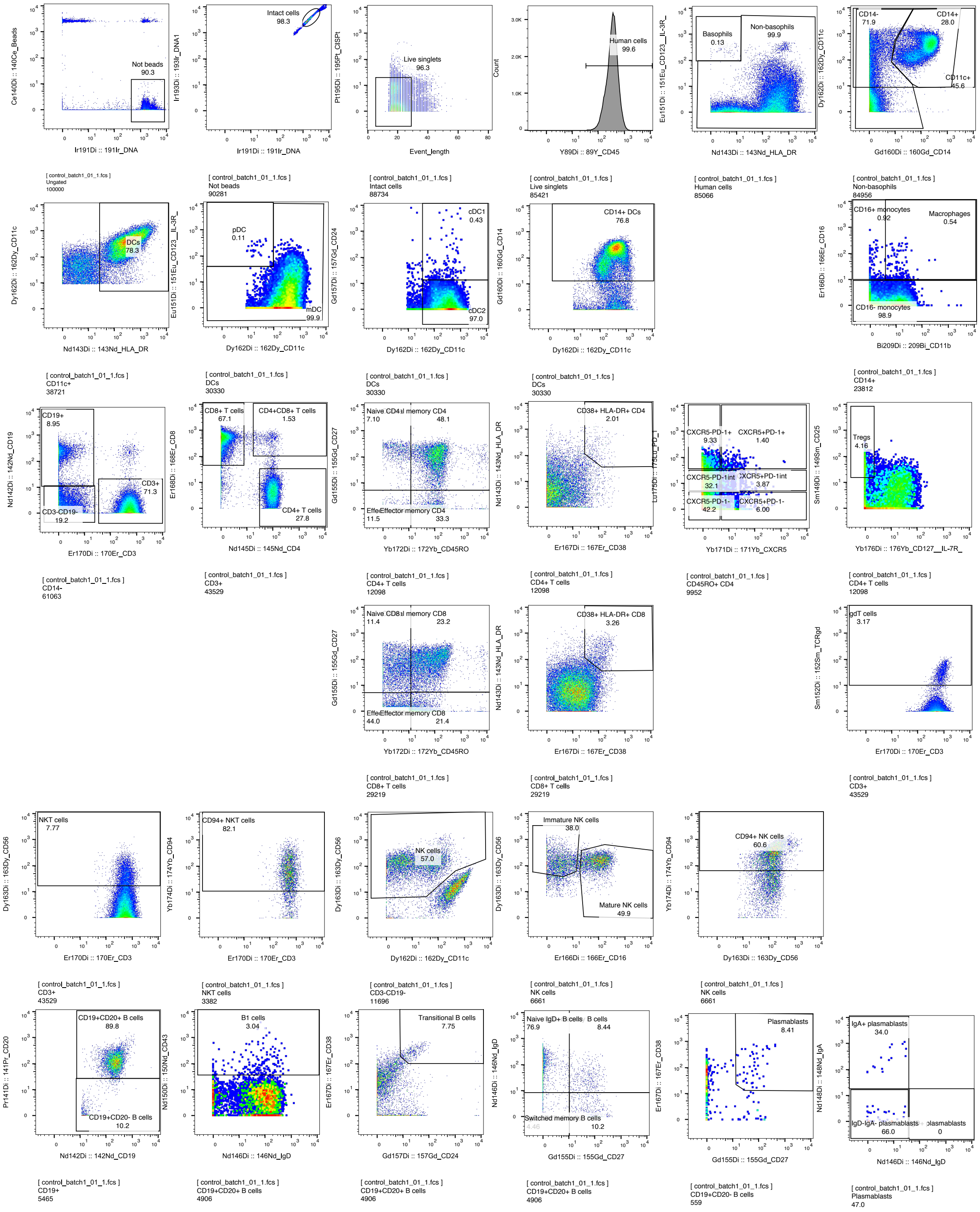

Supplemental Figure 2: Representative core gating scheme utilized for cohort 2 blood and tissue samples. Further subpopulations defined by positive or negative expression of additional markers are not shown.

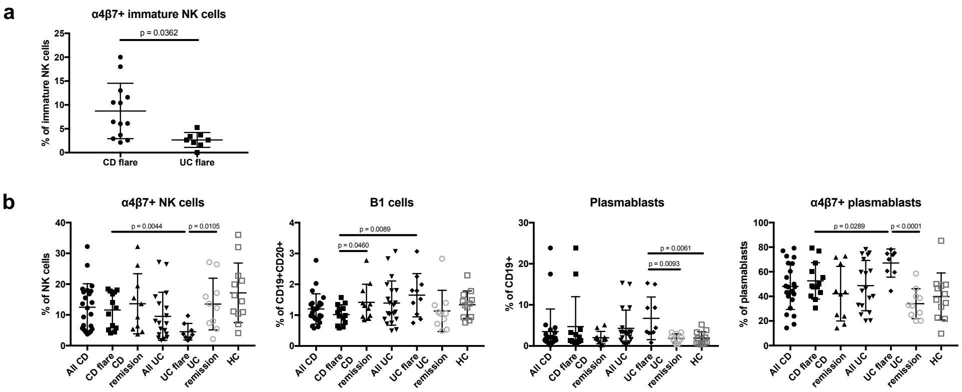

Supplemental Figure 3. Additional blood-based signatures distinguish CD and UC disease groups. (A) $\alpha4\beta7^+$ immature NK cells distinguished CD flare (N=13) and UC flare (N=8) samples. Statistics: BH FDR-corrected unpaired two-tailed Student's T-test using Morpheus (see Methods; t=3.57). (B) Features that distinguished samples by disease state were identified using hypothesis-driven tests. Statistics: unpaired two-tailed Student's T-test ($\alpha4\beta7^+$ NK cells: CD flare vs. UC flare, t=3.23, df=19; UC flare vs. remission, t=2.90, df=16. B1 cells: CD flare vs. UC flare, t=2.89, df=21; CD flare vs. remission, t=2.12, df=22. Plasmablasts: UC flare vs. HC, t=3.06, df=20; UC flare vs. remission, t=2.91, df=18. $\alpha4\beta7^+$ plasmablasts: CD flare vs. UC flare, t=2.36, df=19; UC flare vs. remission, t=5.88, df=16). Sample sizes: all CD=24 (23 for $\alpha4\beta7^+$ NK cells and $\alpha4\beta7^+$ plasmablasts); CD flare=13; CD remission=11 (10 for $\alpha4\beta7^+$ NK cells and $\alpha4\beta7^+$ plasmablasts); all UC=20 (18 for $\alpha4\beta7^+$ NK cells and $\alpha4\beta7^+$ plasmablasts); UC flare=10 (8 for $\alpha4\beta7^+$ NK cells and $\alpha4\beta7^+$ plasmablasts); UC remission=10; HC=12. Center lines=mean; whiskers=standard deviation. All data was generated by mass cytometry. Source data are provided as a Source Data file.

a

Subject ID	Disease diagnosis	Clinical flare or remission	Sex	Age	HBI (CD) or Partial Mayo (UC) disease activity score	Disease localization	Disease phenotype	Perianal disease	Extra-intestinal manifestations	Age at onset	Disease duration (years)	IBD medications	Notes on clinical history
221	CD	R	M	31	2	ileocolonic	Stricturing	N	Y	18	13	5-ASA, MTX, α4β7 antagonist	
238	CD	R	F	28	5	ileocolonic	Stricturing	N	Y	10	18	6-MP, TNF antagonist	
270	CD	R	M	67	0	ileal	Fistulizing	N	N	58	9	MTX, TNF antagonist	
188*	CD	R	F	53	0	ileal	Inflammatory	N	N	48	5	5-ASA	
217	UC	R	M	49	0	pan			N	33	16	5-ASA, 5-ASA enema	
222	UC	R	M	28	0	proctitis			Y	11	17	5-ASA, 5-ASA enema	
190*	UC	R	F	44	1	left-sided			N	26	18	5-ASA, 5-ASA enema	
280	HC		M	43									
289	HC		M	33									
207*	HC		F	63									

b

Marker	Fluorochrome	Source	Clone	Catalog No.
Zombie green	FITC	BioLegend	n/a	423111
CD123	BV421	BioLegend	6H6	306018
CD14	BV510	BioLegend	M5E2	301842
CD11c	PerCP-Cy5.5	BioLegend	Bu15	337210
HLA-DR	BV605	BioLegend	L243	307640
CD19	PE	BioLegend	HIB19	302208
CD3	APC	BioLegend	UCHT1	300412
CD20	BV711	BioLegend	2H7	302342

c

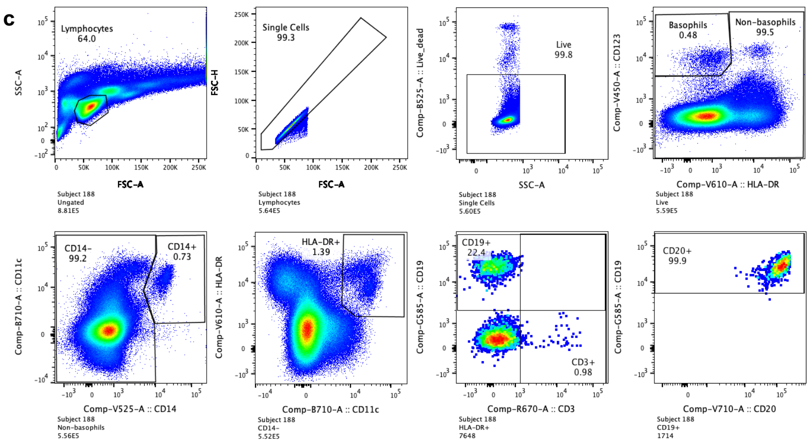

d

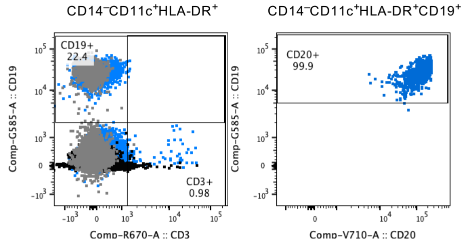

e **f**

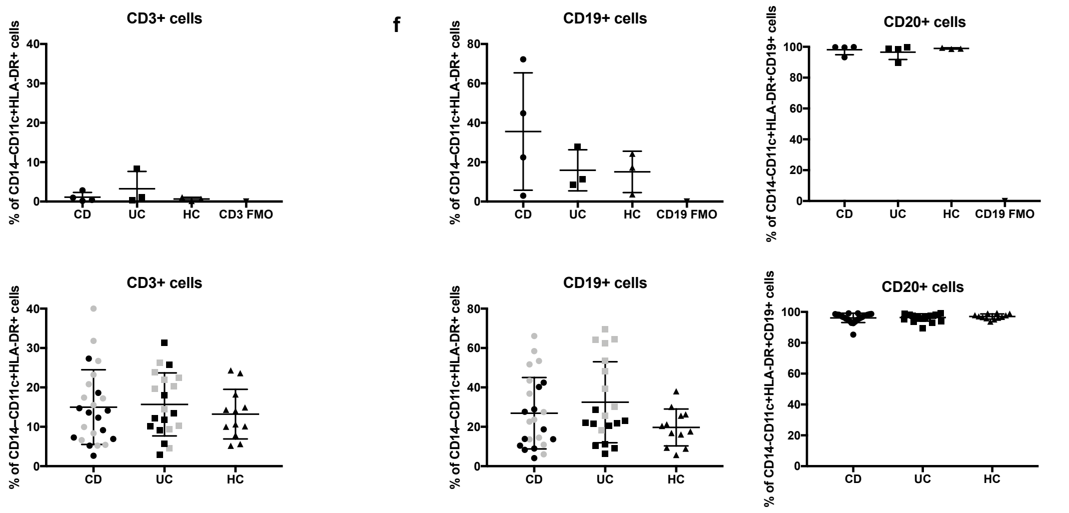

Supplemental Figure 4. Flow cytometry validation of non-canonical leukocyte subsets identified by mass cytometry. (A) Demographic and clinical characteristics of subjects whose samples were utilized for flow cytometry studies in addition to remaining aliquots of samples from subjects in cohorts 1 and 2. All samples consisted of blood PBMCs only. All clinical data reflects the time of sample collection. (CD=Crohn's Disease; UC=ulcerative colitis; HC=healthy control; F/R=flare/remission; F/M=female/male; Y/N=yes/no; 6-MP=6-mercaptopurines; 5-ASA=5-aminosalicylates; MTX=methotrexate). *=subjects also in cohort 1. (B) Antibody panel utilized for samples analyzed by flow cytometry. (C) Flow cytometry gating scheme for a representative sample adapted from the mass cytometry gating scheme in Supplemental Fig. 1. (D) For gates used to define CD3⁺ and CD19⁺ cells from CD14⁻CD11c⁺HLA-DR⁺ cells (left) and CD20⁺ cells from CD14⁻CD11c⁺HLA-DR⁺CD19⁺ cells (right), fluorescence minus one (FMO) controls represent one sample comprised of PBMCs from subjects 188, 190, 207, and 270 in equal parts. Dot color key: blue=subject 188 (CD); black=CD19 FMO control; gray=CD3 FMO control. (E and F) Identification of CD3⁺ cells from CD14⁻CD11c⁺HLA-DR⁺ cells (D), as well as CD19⁺ cells from CD14⁻CD11c⁺HLA-DR⁺ cells and CD20⁺ cells from CD14⁻CD11c⁺HLA-DR⁺CD19⁺ cells (E) by flow cytometry (top row) and mass cytometry (bottom row). Data points for samples from subjects in disease flare are indicated in gray. Sample numbers: CD=4 (flow cytometry), 24 (mass cytometry); UC=3 (flow cytometry), 20 (mass cytometry); HC=3 (flow cytometry), 12 (mass cytometry). Center lines=mean; whiskers=standard deviation. Source data are provided as a Source Data file.

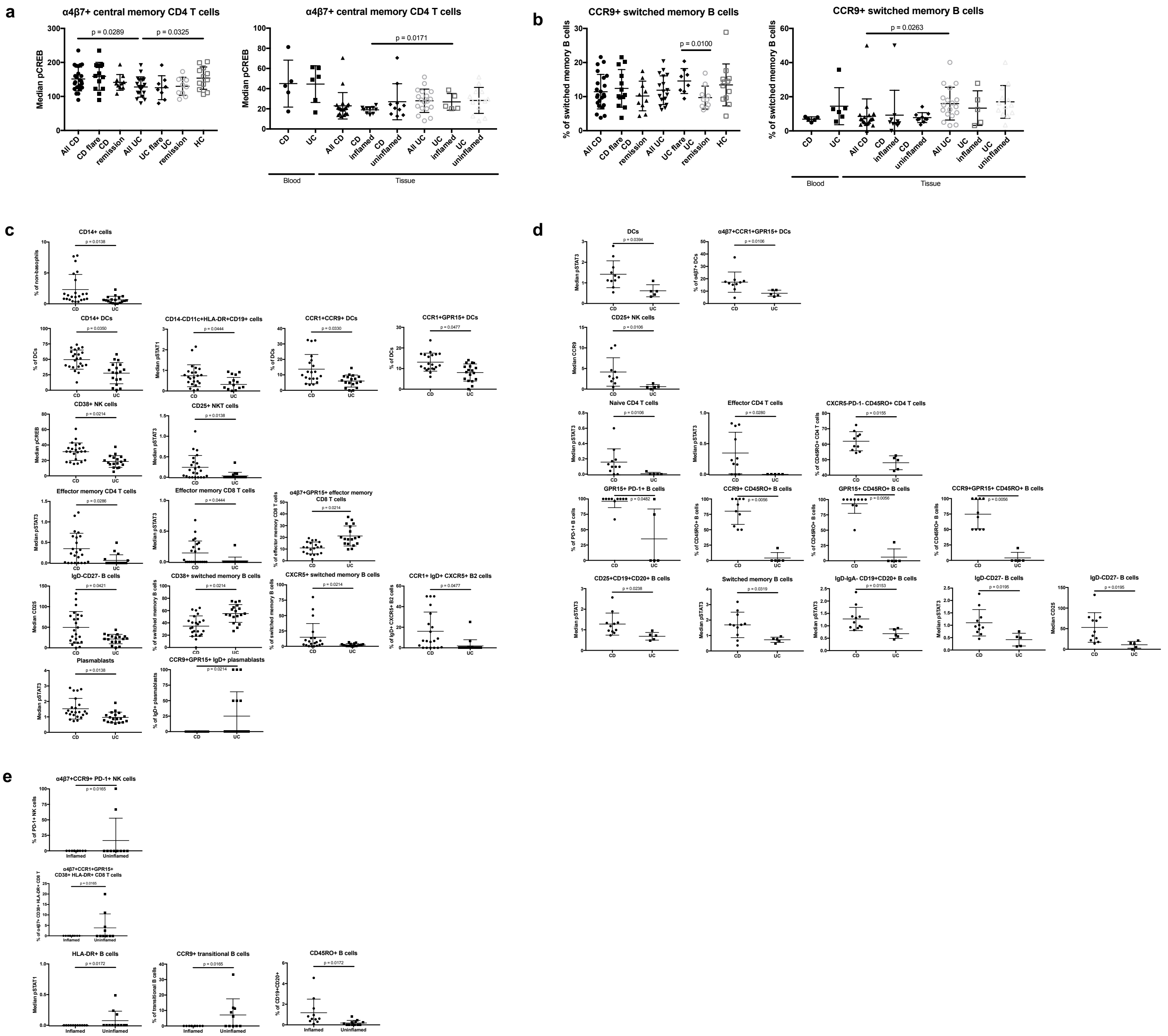

Supplemental Figure 5. Tissue leukocytes distinguish disease and inflammation states. (A and B) Blood-tissue correlates (Fig. 3E) distinguish disease and inflammation states. Statistics: unpaired two-tailed Student's T-test (α4β7⁺ central memory CD4 T cells: all CD vs. all UC, t=2.27, df=39; all UC vs. HC, t=2.25; df=28; CD inflamed vs. UC inflamed tissue, t=2.73, df=13. CCR9⁺ switched memory B cells: UC flare vs. remission, t=2.92, df=16; all CD vs. all UC tissue, t=2.32, df=36). Sample numbers (left): all CD=23; CD flare=13; CD remission=10; all UC=18; UC flare=8; UC remission=10; HC=12. Sample numbers (right): CD blood=5; UC blood=6; all CD tissue=20; CD inflamed tissue=10; CD uninflamed tissue=10; all UC tissue=18; UC inflamed tissue=5; UC uninflamed tissue=13. (C) Features distinguished total CD and UC tissues (Fig. 4B). Statistics: BH FDR-corrected unpaired two-tailed Student's T-test using Morpheus (see Methods; Fig. 4B for t-statistics). Sample sizes: CD=23 (20 for CCR1⁺CCR9⁺ DCs, CCR1⁺GPR15⁺ DCs, α4β7⁺GPR15⁺ effector memory CD8 T cells, CXCR5⁺ switched memory B cells, CCR1⁺ IgD⁺ CXCR5⁺ B2 cells, and CCR9⁺GPR15⁺ IgD⁺ plasmablasts) and UC=18. (D) Features distinguished inflamed CD and UC tissues (Fig. 4C). Statistics: BH FDR-corrected unpaired two-tailed Student's T-test using Morpheus (see Methods; Fig. 4C for t-statistics). Sample sizes: CD=11 (10 for α4β7⁺CCR1⁺GPR15⁺ DCs, CD25⁺ NK cells, CXCR5⁻PD-1⁻ CD45RO⁺ CD4 T cells, GPR15⁺ PD-1⁺ B cells, CCR9⁺ CD45RO⁺ B cells, and CCR9⁺GPR15⁺ CD45RO⁺ B cells) and UC=5. (E) Features distinguished inflamed and uninflamed CD tissues (Fig. 4D). Statistics: BH FDR-corrected unpaired two-tailed Student's T-test using Morpheus (see Methods; Fig. 4D for t-statistics). Sample sizes: inflamed=11 (10 for α4β7⁺CCR9⁺ PD-1⁺ NK cells, α4β7⁺CCR1⁺GPR15⁺ CD38⁺ HLA-DR⁺ CD8 T cells, and CCR9⁺ transitional B cells) and uninflamed=12 (10 for α4β7⁺CCR9⁺ PD-1⁺ NK cells, α4β7⁺CCR1⁺GPR15⁺ CD38⁺ HLA-DR⁺ CD8 T cells, and CCR9⁺ transitional B cells). Center lines=mean; whiskers=standard deviation. All data was generated by mass cytometry. Source data are provided as a Source Data file.

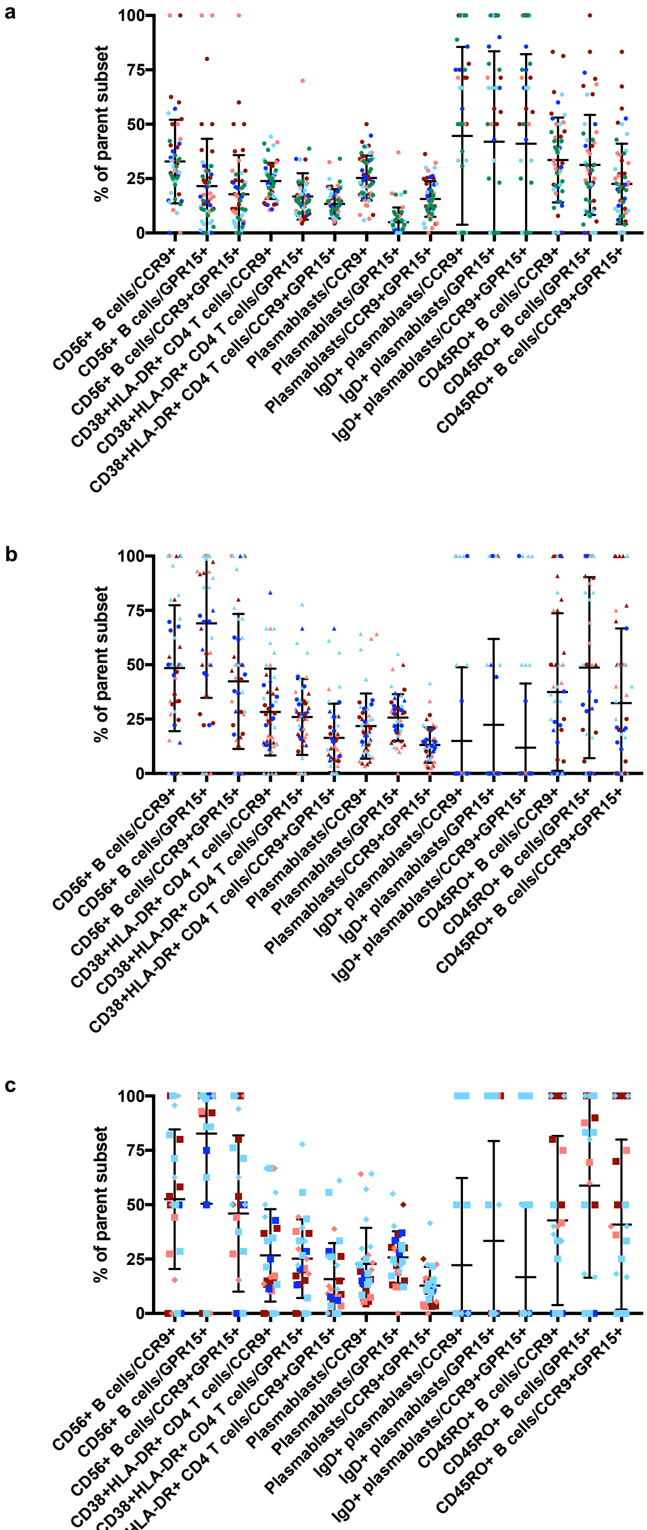

Supplemental Figure 6. CCR9$^+$GPR15$^+$ cells are present across samples and tissues but less abundant than single positive cells. (A) Double positive cells identified in the study and corresponding single positive cells in blood samples from cohort 1 (CD flare=13, dark red; CD remission=10, light red; UC flare=8, dark blue; UC remission=10, light blue; HC=12, green). (B) Double positive cells identified in the study and corresponding single positive cells in blood and tissue samples from cohort 2 (CD blood=5, dark red; inflamed CD tissue=10, dark red; uninflamed CD tissue=10, light red; UC blood=6, dark blue; UC inflamed tissue=5, dark blue; UC uninflamed tissue=13, light red; tissue=triangle; blood=circle). (C) Double positive cells identified in the study and corresponding single positive cells in ileal and colonic tissue from cohort 2 (CD inflamed tissue=7, dark red; CD uninflamed tissue=6, light red; UC inflamed tissue=3, dark blue; UC uninflamed tissue=11, light blue; ileum=10, diamond; colon=16, square). Statistics: center lines=mean; whiskers=standard deviation. All data was generated by mass cytometry. Source data are provided as a Source Data file.

a

Medication	CD (N=30)	UC (N=26)
No medication	5	1
TNF antagonist	15	7
Glucocorticoids	9	11
Glucocorticoid enema	0	1
5-ASA	6	21
5-ASA enema	0	10
6-MP	11	6
$\alpha 4\beta 7$ antagonist	2	2
IL-12/23 antagonist	3	0
Methotrexate	6	1

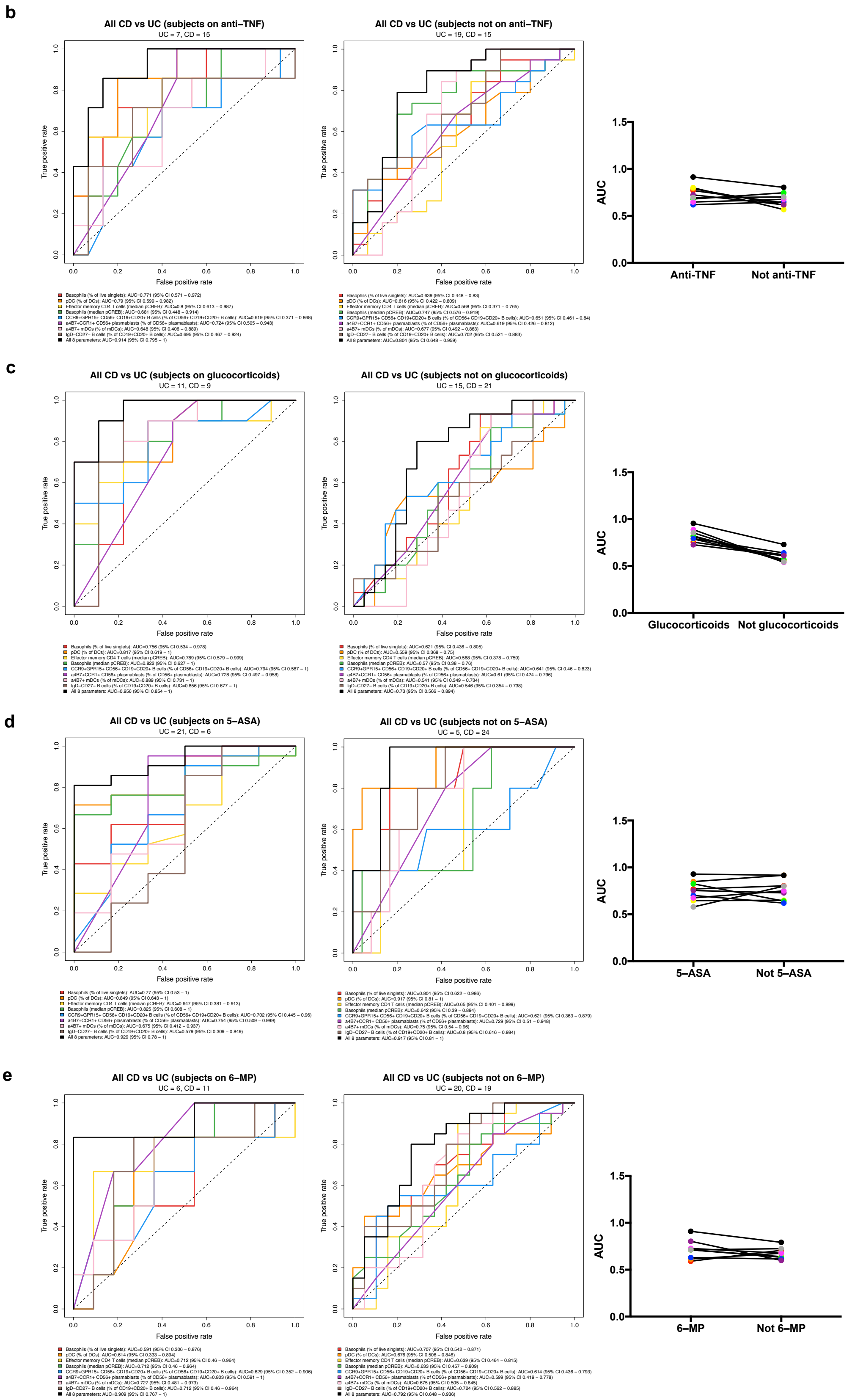

Supplemental Figure 7. Accounting for medication improves disease group classification. (A) Numbers of subjects from cohort 1 on each medication, regardless of other combination therapy. Subjects administered 5-ASA by enema were also on oral 5-ASA, and subjects administered glucocorticoids by enema were also on oral glucocorticoids. The same generalized linear model (GLM) previously created using eight parameters to classify cohort 1 CD versus UC samples (Fig. 5B) was used for classification of samples from all subjects on (left) or not on (right) TNF antagonists (B), glucocorticoids (C), 5-ASA (D), or 6-MP (E). Corresponding receiver operating characteristic (ROC) curves are shown with comparisons to single feature models. UC was used as baseline for the purposes of the GLMs, such that a true positive indicates correct classification of a CD sample. To the right of ROC curves is a representation of the corresponding AUCs for each single and multi-parameter model (same color key as ROC curves) paired by calculation based on subjects on or off of the respective medication. Statistics: generalized linear models were constructed using *glm* in R (see Methods). Intercepts and parameter coefficients for the model described in Fig. 5B are provided in Supplemental Table 4. (anti-TNF=TNF antagonists; 5-ASA=5-aminosalicylates; 6-MP=6-mercaptopurines). Source data are provided as a Source Data file.

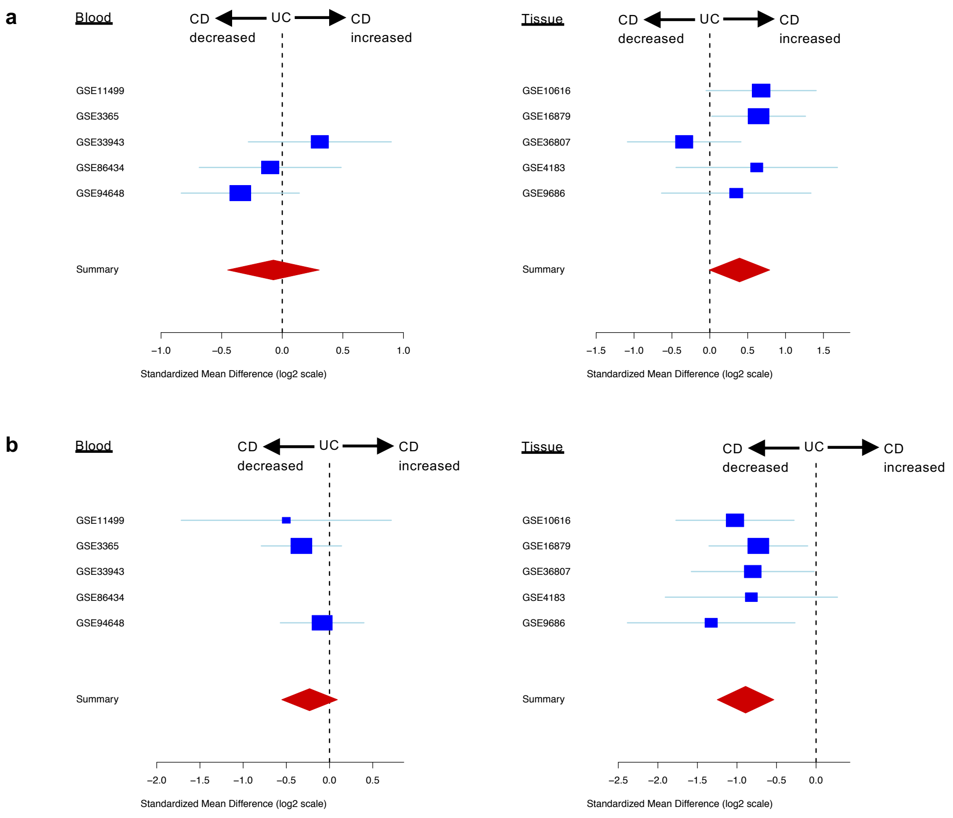

Supplemental Figure 8. Gene expression deconvolution analysis reveals consistent trends with mass cytometry data. Gene expression deconvolution analyses using publicly available microarray datasets revealed relative trends for (A) basophils and (B) plasma cells that were consistent with CyTOF data presented in the study. Each row represents a dataset with overall effect size of the cell type frequency calculated (dark blue boxes); confidence intervals were also computed (light blue line). See Methods for details on deconvolution. Datasets were obtained using the NCBI GEO database (https://www.ncbi.nlm.nih.gov/geo/). Source data are provided as a Source Data file.

Subject ID	Disease diagnosis	Clinical flare or remission	Sex	Age	HBI (CD) or Partial Mayo (UC) disease activity score	Disease localization	Disease phenotype	Perianal disease	Extra-intestinal manifestations	Age at onset	Disease duration (years)	IBD medications	Notes on clinical history
118	CD	F	F	48	6	ileocolonic	Stricturing	N	N	19	30	TNF antagonist, methotrexate, glucocorticoids	
119	CD	F	F	65	7	ileocolonic	Stricturing	N	N	27	39	TNF antagonist, methotrexate	
124	CD	F	M	23	5	ileal	Stricturing	N	N	5	18	TNF antagonist, methotrexate, glucocorticoids	
127	CD	F	M	39	5	ileocolonic	Fistulizing	N	N	30	10	6-MP, glucocorticoids	
128	CD	F	M	23	2	ileal	Stricturing	N	N	5	18	methotrexate, glucocorticoids	Repeat of 124 after 1 month
129	CD	F	M	43	7	ileocolonic	Stricturing, fistulizing	Y	N	17	26	IL-12/23 antagonist, 6-MP, glucocorticoids	
138	CD	R	F	39	2	ileocolonic	Stricturing	N	N	29	10	TNF antagonist, 5-ASA	
143	CD	R	M	31	2	ileal	Stricturing	Y	N	14	17	TNF antagonist, methotrexate	
146	CD	R	F	51	0	colonic	Fistulizing	N	N	21	30	none	
152	CD	R	M	40	2	colonic	Stricturing	N	N	6	34	TNF antagonist, 5-ASA, 6-MP	
158	CD	F	M	42	5	ileocolonic	Inflammatory	Y	N	8	34	TNF antagonist, 5-ASA, 6-MP, glucocorticoids	
160	CD	F	F	25	3	ileocolonic	Fistulizing	Y	Y	16	9	IL-12/23 antagonist, 5-ASA	
167	CD	R	F	35	7	ileocolonic	Fistulizing	Y	Y	16	19	6-MP	
171	CD	R	F	41	0	colonic	Inflammatory	N	N	31	10	TNF antagonist	
177	CD	R	F	35	0	ileocolonic	Fistulizing	N	Y	16	30	none	
178	CD	F	M	27	5	ileocolonic	Fistulizing	Y	Y	27	0.08	none	
184	CD	F	M	47	1	colonic	Fistulizing	Y	N	39	8	TNF antagonist	
185	CD	F	F	57	3	ileal	Inflammatory	N	N	55	2	IL-12/23 antagonist, glucocorticoids	
187	CD	R	F	23	0	ileocolonic	Inflammatory	N	Y	10	13	6-MP	Past history of stricturing disease
188	CD	R	F	53	0	ileal	Inflammatory	N	N	48	5	5-ASA	
189	CD	R	F	33	0	ileocolonic	Inflammatory	N	N	13	10	TNF antagonist, 5-ASA, 6-MP	
196	CD	R	M	27	0	ileocolonic	Inflammatory	N	N	13	14	α4β7 antagonist	
202	CD	F	F	51	4	ileocolonic	Stricturing, fistulizing	N	N	51	0.25	TNF antagonist, 6-MP, glucocorticoids	
205	CD	F	M	35	4	ileocolonic	Inflammatory	N	Y	34	1	TNF antagonist, methotrexate, glucocorticoids	
117	UC	F	F	28	7	pan			N	12	16	TNF antagonist, glucocorticoids	
120	UC	F	F	21	9	pan			N	16	5	5-ASA, glucocorticoids	
121	UC	F	F	68	7	left-sided			N	40	28	TNF antagonist, glucocorticoids, glucocorticoid enema	
122	UC	F	F	36	7	left-sided			N	34	2	α4β7 antagonist, glucocorticoids	
123	UC	F	M	60	8	left-sided			N	31	29	5-ASA, 6-MP, glucocorticoids	
139	UC	R	F	37	0	pan			N	20	17	none	
140	UC	R	F	75	1	pan			N	60	15	5-ASA, 5-ASA enema	
141	UC	R	F	27	0	proctitis			N	25	2	5-ASA	
145	UC	R	F	37	3	left-sided			N	27	10	5-ASA, 6-MP	
159	UC	F	M	31	6	pan			N	28	3	TNF antagonist, 5-ASA, glucocorticoids, 5-ASA enema	
161	UC	R	M	33	2	pan			N	29	4	5-ASA, 5-ASA enema	
168	UC	R	F	35	2	pan			Y	24	11	TNF antagonist, 5-ASA, 5-ASA enema	
170	UC	R	M	71	2	pan			N	29	42	5-ASA, 6-MP	
175	UC	R	M	45	2	pan			Y	17	28	TNF antagonist, 5-ASA, 6-MP	
180	UC	F	M	58	5	pan			N	55	3	5-ASA, glucocorticoids	
190	UC	R	F	44	1	left-sided			N	26	18	5-ASA, 5-ASA enema	
191	UC	F	F	19	8	pan			N	18	1	5-ASA, glucocorticoids	
198	UC	F	F	42	6	left-sided			N	40	2	5-ASA, 5-ASA enema, glucocorticoids	
199	UC	R	M	35	2	left-sided			N	32	3	5-ASA, 5-ASA enema	
204	UC	F	F	19	5	pan			N	18	1	α4β7 antagonist, 5-ASA, glucocorticoids, 5-ASA enema	Repeat of 191 after 1 month
154	HC		M	35									
155	HC		M	34									
165	HC		M	63									
172	HC		M	54									
192	HC		M	41									
193	HC		F	63									
206	HC		M	56									
207	HC		F	63									
208	HC		M	47									
210	HC		M	57									
211	HC		F	24									
212	HC		F	33									

Supplemental Table 1. Demographic and clinical characteristics of subjects in cohort 1. Cohort 1 contained blood samples only. All clinical data reflects the time of sample collection. (CD=Crohn's Disease; UC=ulcerative colitis; HC=healthy control; F/R=flare/remission; F/M=female/male; Y/N=yes/no; 6-MP=6-mercaptopurines; 5-ASA=5-aminosalicylates).

Sample ID	Disease diagnosis	Sex	Age	Sample type	Biopsy state	Disease localization	HBI (CD) or Partial Mayo (UC) disease activity score	Disease phenotype	Perianal disease	Extra-intestinal manifestations	Age at onset	Disease duration (years)	IBD medications	Notes on endoscopic findings
239_0	CD	F	22	PBMCs		ileocolonic	6	Stricturing	N	Y	19	3	none	endoscopic mildly active disease
239_1	CD			sigmoid colon	inflamed	ileocolonic		Stricturing	N	Y				mild inflammation
239_2	CD			rectum	inflamed	ileocolonic		Stricturing	N	Y				mild inflammation
239_3	CD			ileo-cecal valve	inflamed	ileocolonic		Stricturing	N	Y				severe stricture, pseudopolyps
239_4	CD			right colon	uninflamed	ileocolonic		Stricturing	N	Y				
240_0	CD	F	48	PBMCs		colonic	0	Fistulizing	N	Y	37	11	TNF antagonist, 6-MP	endoscopic remission or very mildly active disease
240_1	CD			rectum	uninflamed	colonic		Fistulizing	N	Y				
240_2	CD			terminal ileum	uninflamed	colonic		Fistulizing	N	Y				
240_3	CD			left colon	inflamed	colonic		Fistulizing	N	Y				pseudopolyps, scarring
240_4	CD			sigmoid colon	inflamed	colonic		Fistulizing	N	Y				scarring
243_0	CD	F	65	PBMCs		ileocolonic	1	Stricturing, fistulizing	N	Y	48	17	TNF antagonist	endoscopic moderate disease
243_1	CD			right colon	inflamed	ileocolonic		Stricturing, fistulizing	N	Y				inflamed
243_2	CD			left colon	inflamed	ileocolonic		Stricturing, fistulizing	N	Y				inflamed stricture, ulcerations
243_3	CD			terminal ileum	uninflamed	ileocolonic		Stricturing, fistulizing	N	Y				
243_4	CD			duodenum	uninflamed	ileocolonic		Stricturing, fistulizing	N	Y				
249_0	CD	F	40	PBMCs		ileocolonic	2	Fistulizing	Y	N	24	16	none	endoscopic remission or very low inflammation
249_1	CD			terminal ileum	uninflamed	ileocolonic		Fistulizing	Y	N				
249_2	CD			rectum	uninflamed	ileocolonic		Fistulizing	Y	N				
249_3	CD			recto-sigmoid	inflamed	ileocolonic		Fistulizing	Y	N				mild inflammation, pseudopolyps
249_4	CD			ileocolonic anastamosis	inflamed	ileocolonic		Fistulizing	Y	N				anastamosis
249_5	CD			right colon	uninflamed	ileocolonic		Fistulizing	Y	N				
252_0	CD	M	60	PBMCs		ileocolonic	0	Stricturing	N	N	52	7	α4β7 antagonist, 6-MP	endoscopic mild to moderate small bowel disease
252_1	CD			ileum	inflamed	ileocolonic		Stricturing	N	N				stricture, large ulcer
252_2	CD			rectum	uninflamed	ileocolonic		Stricturing	N	N				
252_3	CD			duodenum	uninflamed	ileocolonic		Stricturing	N	N				
255_0	CD	F	26	PBMCs		ileal	0	Stricturing, fistulizing	N	N	25	1	TNF antagonist, 6-MP	endoscopic remission
255_1	CD			distal ileum	uninflamed	ileal		Stricturing, fistulizing	N	N				
255_2	CD			rectum	uninflamed	ileal		Stricturing, fistulizing	N	N				
255_3	CD			distal ileum	inflamed	ileal		Stricturing, fistulizing	N	N				pseudopolyps, stricture
234_0	UC	M	34	PBMCs		pan	0			N	24	10	TNF antagonist, 5-ASA, methotrexate	endoscopic remission
234_1	UC			terminal ileum	uninflamed	pan				N				
234_2	UC			right colon	uninflamed	pan				N				
234_3	UC			recto-sigmoid	uninflamed	pan				N				
235_0	UC	M	60	PBMCs		left	1			Y	43	17	TNF antagonist	endoscopic very mild rectal disease
235_1	UC			rectum	inflamed	left				Y				mild inflammation
235_2	UC			right colon	uninflamed	left				Y				
235_3	UC			terminal ileum	uninflamed	left				Y				
251_0	UC	F	48	PBMCs		left	0			Y	35	13	5-ASA, 5-ASA enema	endoscopic remission
251_1	UC			rectum	uninflamed	left				Y				
251_2	UC			right colon	uninflamed	left				Y				
251_3	UC			terminal ileum	uninflamed	left				Y				
261_0	UC	M	25	PBMCs		left	0			N	21	4	5-ASA, 6-MP	endoscopic remission
261_1	UC			transverse colon	inflamed	left				N				normal/quiescent colitis
261_2	UC			rectum	inflamed	left				N				mild proctitis
261_3	UC			right colon	uninflamed	left				N				
263_0	UC	M	33	PBMCs		pan	0			N	22	11	5-ASA	endoscopic remission
263_1	UC			right colon	uninflamed	pan				N				
263_2	UC			rectum	uninflamed	pan				N				
263_3	UC			terminal ileum	uninflamed	pan				N				
264_0	UC	M	42	PBMCs		pan	3			Y	38	4	5-ASA, 6-MP, glucocorticoids, 5-ASA enema	endoscopic very mild disease
264_1	UC			right colon	inflamed	pan				Y				pseudopolyps
264_2	UC			terminal ileum	uninflamed	pan				Y				
264_3	UC			recto-sigmoid	inflamed	pan				Y				mild inflammation, inflammatory polyp

Supplemental Table 2. Demographic and clinical characteristics of subjects in cohort 2. Cohort 2 contained paired blood and tissue biopsy samples. All clinical data reflects the time of sample collection. All patients in cohort 2 were in clinical remission, although some had endoscopically active disease (see notes). (CD=Crohn's Disease; UC=ulcerative colitis; HC=healthy control; F/R=flare/remission; F/M=female/male; Y/N=yes/no; 6-MP=6-mercaptopurines; 5-ASA=5-aminosalicylates).

a

Metal	Marker	Clone	Source	Catalog No.
140 Ce	Beads	n/a	Fluidigm	201078
141Pr	CD20	2H7	BioLegend, Fluidigm	302343, 201141A
142Nd	CD19	HIB19	Fluidigm	3142001B
143Nd	CD5	UCHT2	Fluidigm	3143007B
144Nd	pPLCγ2 [Y759]	K86-689.37	Fluidigm	3144015A
145Nd	CD4	RPA-T4	Fluidigm	3145001B
146Nd	IgD	IA6-2	Fluidigm	3146005B
147Nd	pSTAT5 [Y694]	47	Fluidigm	3150005A
148Nd	IgA	Polyclonal	Fluidigm	3148007B
149Sm	CD25 (IL-2R)	2A3	Fluidigm	3149010B
150Nd	CD43	84-3C1	Fluidigm	3150006B
151Eu	CD123 (IL-3R)	6H6	Fluidigm	3151001B
152Sm	Akt [S473]	D9E	Fluidigm	3152005A
153Eu	pSTAT1 [Y701]	4a	Fluidigm	3153005A
154Sm	GPR15-PE	SA302A10	BioLegend, Fluidigm	Custom, 201154A
155Gd	CD27	L128	Fluidigm	3155001B
156Gd	p-p38 [T180/Y182]	D3F9	Fluidigm	3156002A
157Gd	CD24	ML-5	BioLegend and Stanford HIMC	311127, custom
158Gd	pSTAT3 [Y705]	4	Fluidigm	3158005A
159Tb	pMAPKAPK2	27B7	Fluidigm	3159010A
160Gd	CD14	M5E2	Fluidigm	3160001B
161Dy	CCR9	L053E8	BioLegend, Fluidigm	358902, 201161A
162Dy	CD11c	Bu15	Fluidigm	3162005B
163Dy	CD56 (NCAM)	NCAM16.2	Fluidigm	3163007B
164Dy	IκBa	L35A5	Fluidigm	3164004A
165Ho	pCREB [S133]	87G3	Fluidigm	3165009A
166Er	CD16	B73.1	Stanford HIMC	Custom
167Er	CD38	HIT2	Fluidigm	3167001B
168Er	CD8α	SK1	Fluidigm	3168002B
169Tm	CCR1	5F10B29	BioLegend, Fluidigm	362902, 201169A
170Er	CD3	UCHT1	Fluidigm	3170001B
171Yb	pERK 1/2 [T202/Y204]	D13.14.4E	Fluidigm	3171010A
172Yb	CD45RO	UCHL1	BioLegend, Fluidigm	304239, 201172A
173Yb	$\alpha4\beta7$	Act1	NIH AIDS Reagent Program, Fluidigm	11718, 201173A
174Yb	HLA-DR	L243	Fluidigm	3174001B
175Lu	pS6	N7548	Fluidigm	3175009A
176Yb	CD127 (IL-7Ra)	A019D5	Fluidigm	3176004B
209Bi	CD11b	ICRF44	Fluidigm	3209003B
191Ir	DNA	n/a	Fluidigm	201192B
193Ir	DNA	n/a	Fluidigm	201192B
195Pt	Cisplatin viability (live/dead)	n/a	Fluidigm	201064

b

Metal	Marker	Clone	Source	Catalog No.
89Y	CD45	HI30	Fluidigm	3089003B
140 Ce	Beads	n/a	Fluidigm	201078
141Pr	CD20	2H7	BioLegend, Fluidigm	302343, 201141A
142Nd	CD19	HIB19	Fluidigm	3142001B
143Nd	HLA-DR	L243	Fluidigm	3143013B
144Nd	pPLCγ2 [Y759]	K86-689.37	Fluidigm	3144015A
145Nd	CD4	RPA-T4	Fluidigm	3145001B
146Nd	IgD	IA6-2	Fluidigm	3146005B
147Nd	pSTAT5 [Y694]	47	Fluidigm	3150005A
148Nd	IgA	Polyclonal	Fluidigm	3148007B
149Sm	CD25 (IL-2R)	2A3	Fluidigm	3149010B
150Nd	CD43	84-3C1	Fluidigm	3150006B
151Eu	CD123 (IL-3R)	6H6	Fluidigm	3151001B
152Sm	TCR$\gamma\delta$	11F2	Fluidigm	3152008B
153Eu	pSTAT1 [Y701]	4a	Fluidigm	3153005A
154Sm	GPR15-PE	SA302A10	BioLegend, Fluidigm	Custom, 201154A
155Gd	CD27	L128	Fluidigm	3155001B
156Gd	p-p38 [T180/Y182]	D3F9	Fluidigm	3156002A
157Gd	CD24	ML-5	BioLegend and Stanford HIMC	311127, custom
158Gd	pSTAT3 [Y705]	4	Fluidigm	3158005A
159Tb	pMAPKAPK2	27B7	Fluidigm	3159010A
160Gd	CD14	M5E2	Fluidigm	3160001B
161Dy	CCR9	L053E8	BioLegend, Fluidigm	358902, 201161A
162Dy	CD11c	Bu15	Fluidigm	3162005B
163Dy	CD56 (NCAM)	NCAM16.2	Fluidigm	3163007B
164Dy	IκBa	L35A5	Fluidigm	3164004A
165Ho	pCREB [S133]	87G3	Fluidigm	3165009A
166Er	CD16	B73.1	Stanford HIMC	Custom
167Er	CD38	HIT2	Fluidigm	3167001B
168Er	CD8α	SK1	Fluidigm	3168002B
169Tm	CCR1	5F10B29	BioLegend, Fluidigm	362902, 201169A
170Er	CD3	UCHT1	Fluidigm	3170001B
171Yb	CXCR5	RF8B2	Fluidigm	3171014B
172Yb	CD45RO	UCHL1	BioLegend, Fluidigm	304239, 201172A
173Yb	$\alpha4\beta7$	Act1	NIH AIDS Reagent Program, Fluidigm	11718, 201173A
174Yb	CD94	HP-3D9	Fluidigm	3174015B
175Lu	PD-1	EH12.2H7	Fluidigm	3175008B
176Yb	CD127 (IL-7Ra)	A019D5	Fluidigm	3176004B
209Bi	CD11b	ICRF44	Fluidigm	3209003B
191Ir	DNA	n/a	Fluidigm	201192B
193Ir	DNA	n/a	Fluidigm	201192B
195Pt	Cisplatin viability (live/dead)	n/a	Fluidigm	201064

Supplemental Table 3. CyTOF panels utilized in the study. (A) CyTOF panel utilized for samples from cohort 1; (B) CyTOF panel utilized for samples from cohort 2.

All CD vs UC (training dataset)	
Intercept	-0.3587230
Basophils (% of live singlets)	-1.0432548
pDC (% of DCs)	0.2258415
Effector memory CD4 T cells (median pCREB)	-0.5494137
Basophils (median pCREB)	-0.3559057
CCR9+GPR15+ CD56+ CD19+CD20+ B cells (% of CD56+ CD19+CD20+ B cells)	-0.6370560
$\alpha 4\beta 7$+CCR1+ CD56+ plasmablasts (% of CD56+ plasmablasts)	-0.7015127
$\alpha 4\beta 7$+ mDCs (% of mDCs)	-0.2405979
IgD–CD27– B cells (% of CD19+CD20+ B cells)	0.7702076

Supplemental Table 4. Parameters of generalized linear models used for disease classifications. Intercepts and coefficients for each parameter of the generalized linear models presented in Fig. 5B are shown for the corresponding model. Statistics: models were constructed using *glm* in R (see Methods).

	Cohort 1			Cohort 2		
	**df**	**F-value**	**P-value**	**df**	**F-value**	**P-value**
**Basophils (% of live singlets)**						
Age	1	0.089	0.766	1	0.391	0.549
Sex	1	0.003	0.957	1	0.045	0.837
Age:Sex	1	2.015	0.164	1	0.692	0.430
**pDC (% of DCs)**						
Age	1	0.000	0.985	1	0.067	0.803
Sex	1	0.017	0.898	1	0.141	0.717
Age:Sex	1	1.097	0.301	1	0.050	0.829
**Effector memory CD4 T cells (median pCREB)**						
Age	1	0.314	0.578	1	0.014	0.908
Sex	1	0.312	0.580	1	0.851	0.383
Age:Sex	1	0.051	0.823	1	0.460	0.460
**Basophils (median pCREB)**						
Age	1	0.026	0.873	1	0.251	0.630
Sex	1	0.085	0.772	1	0.208	0.661
Age:Sex	1	2.352	0.133	1	1.670	0.232
**CCR9+GPR15+ CD56+ CD19+CD20+ B cells (% of CD56+ CD19+CD20+ B cells)**						
Age	1	0.058	0.811	1	0.176	0.886
Sex	1	0.091	0.764	1	4.378	0.070
Age:Sex	1	0.378	0.542	1	0.007	0.936
**a4B7+CCR1+ CD56+ plasmablasts (% of CD56+ plasmablasts)**						
Age	1	0.471	0.496	1	3.166	0.113
Sex	1	0.715	0.403	1	3.663	0.092
Age:Sex	1	0.198	0.659	1	2.892	0.127
**a4B7+ mDCs (% of mDCs)**						
Age	1	0.019	0.890	1	4.175	0.075
Sex	1	0.308	0.582	1	4.781	0.060
Age:Sex	1	1.767	0.191	1	2.350	0.164
**IgD-CD27- B cells (% of CD19+CD20+ B cells)**						
Age	1	0.001	0.976	1	2.120	0.183
Sex	1	0.333	0.567	1	1.672	0.232
Age:Sex	1	2.534	0.119	1	0.259	0.624

Supplemental Table 5. Analysis of covariance (ANCOVA) for age and sex with each parameter used in the GLM. ANCOVA was performed using the *aov* package in R (see Methods) for age and sex with each of the eight parameters used in the GLM (see Figure 5B). Statistics: df, F-, and P-values are reported in the table. Data is from cohorts 1 and 2; N=30 (CD) and 26 (UC).

Cell subset	Lineage	Gating	Description	References
B1 cells	B cell	CD14–CD19+CD20+CD43+IgDvar	Innate-like; low frequency in blood; high frequency in peritoneum; CD5+ B1a subset is protective in mouse colitis	1
CD25+ CD19+CD20+ B cells	B cell	CD14–CD19+CD20+CD25+	Regulatory subset; secretes IL-10 and TGF-β; abnormal frequency reported in UC	2-5
CD38+ switched memory B cell	B cell	CD14–CD19+CD20+IgD–CD27+CD38+	Activated cells; similar to germinal center phenotype; about half of CD27+ B cells are CD38+, most of which are CD24+ and could include transitional B cells but unlikely since IgD-	6
CD45RO+ B cells	B cell	CD14–CD19+CD20+CD45RO+	Proposed biomaker for CD	7
CD56+ B cells	B cell	CD14–CD19+CD20+CD56+	CD56+ B cells reported in lymphoma; may indicate extreme activation	8
CD56+ plasmablasts	B cell	CD14–CD19+CD20-CD27+CD38+CD56+	CD56+ B cells reported in lymphoma; may indicate extreme activation	8
CXCR5+ switched memory B cells	B cell	CD14–CD19+CD20+IgD–CD27+CXCR5+	Expressed on most mature circulating B cells; responsible for migration to secondary lymphoid organs; associated with GC B cells; CXCR5+ memory B cells decreased in RA and SLE	9
HLA-DR+ B cells	B cell	CD14–CD19+CD20+HLA-DR+	HLA-DR expressed by most B cells; numerous studies demonstrating HLA-DR and HLA-DQ associations with IBD	10
IgD–CD27– B cells	B cell	CD14–CD19+CD20+IgD–CD27–	Larger and more granular than IgD+CD27– naïve B cells; class-switched and somaticlly hypermutated, suggesting antigenic selection; expansded in SLE, HIV and rotavirus infection	11-16
IgD–IgA– B cells	B cell	CD14–CD19+CD20+IgD–IgA–	Gating strategy used to identify likely IgG+ B cells without staining for IgG	
IgD+ CXCR5+ B2 cells	B cell	CD14–CD19+CD43–CXCR5+IgD+	Non-B1 cells that express IgD and CXCR5 (see above)	
PD-1+ B cells	B cell	CD14–CD19+CD20+PD-1+	Regulates B cell activation; expressed on naïve and memory but not GC B cells	17
Switched memory B cells	B cell	CD14–CD19+CD20+IgD–CD27+	Memory B cells that lack IgD expression and thus express IgA, IgG, or IgE	18
Transitional B cells	B cell	CD14–CD19+CD20+CD24+CD38+	Newly formed B cells from the bone marrow that emigrate into circulation and/or secondary lymphoid organs	18
CD14+ DCs	DC	CD11c+HLA-DR+CD14+	Found in tissues; more monocyte-like; previously described as interstitial DCs	19
CD19+CD11c+HLA-DR+	DC	CD11c+HLA-DR+CD19+	May include B cells and minor DC population uniquely responsive to B7 ligation via IFNα-mediated STAT1 activation; DCs may play regulatory role	20
CD3+CD11c+HLA-DR+	DC	CD11c+HLA-DR+CD3+	May include highly activated T cells and/or minor DC population; most CD3+ DCs also express CD8	21
HLA-DRlo DCs	DC	CD11c+HLA-DRlo	Fraction of DCs that express less HLA-DR but are still positive for this marker; HLA-DR can be downregulated late after DC activation; immature DCs also re-endocytose and degrade peptide-MHC complexes faster than activated DCs; diminished HLA-DR expression by DCs in neonates also associated with impaired antimicrobial defense	22,23
CD11c+ NK cells	NK cell	CD14–CD3–CD19–CD56+CD11c+	NK cells expressing high levels of CD11c associated with higher HLA-DR expression in PBMCs from MS patients; CD11c upregulated by IL-15; associated with clinical relapse in MS patients	24
CD16– NK cells	NK cell	CD14–CD3–CD19–CD56+CD16-	Cytokine activation leads to decreased CD16 expression and is correlated with increased IFNγ production; expanded in blood of patients with metastatic melanoma	25,26
CD16+ NK cells	NK cell	CD14–CD3–CD19–CD56+CD16+	CD16 expressed by most CD56dim peripheral NK cells; provides potent signal to NK cells upon binding antibody coated cells	25
CD25+ NK cells	NK cell	CD14–CD3–CD19–CD56+CD25+	CD25 expression associated with activation of NK cells; increase reported in IBD	27
CD38+ NK cells	NK cell	CD14–CD3–CD19–CD56+CD38+	CD38 signaling in NK cells associated with activation and cytotoxicity; required for antitumor effects	28-30
CD38+CD43+ NK cells	NK cell	CD14–CD3–CD19–CD56+CD38+CD43+	See notes on CD38+ NK cells and CD43+ NK cells	
CD43+ NK cells	NK cell	CD14–CD3–CD19–CD56+CD43+	CD43 signaling in NK cells associated with activation, chemokine reease, and cytotoxic activity	31,32
Immature NK cells	NK cell	CD14–CD3–CD19–CD16–CD56hi	Associated with greater proliferative capacity and cytokine production	33
Mature NK cells	NK cell	CD14–CD3–CD19–CD16+CD56lo	Associated with greater cytolytic activity	33
PD-1+ NK cells	NK cell	CD14–CD3–CD19–CD56+PD-1+	Increased expression of PD-1 on NK cells associated with impaired NK-cell-mediated anti-tumor functions; indicative of poor prognosis in digestive cancers	34
CD25+ NKT cells	NKT cell	CD14–CD3+CD56+CD25+	CD25 expression associated with activation of NKT cells; increase reported in IBD	27
CD38+ NKT cells	NKT cell	CD14–CD3+CD56+CD38+	CD38 required for survival or tolerigenic NKT cells in NOD mice	35
CD43+ NKT cells	NKT cell	CD14–CD3+CD56+CD43+	CD43 expression associated with NKT cell activation and maturation	36,37
CD45RO+ NKT cells	NKT cell	CD14–CD3+CD56+CD45RO+	CD45RO expression associated with NKT cell activation and memory phenotype	38
CD38+HLA-DR+ CD4 T cells	T cell	CD14–CD3+CD4+CD38+HLA-DR+	Highly activated phenotype; abundance correlated with levels of LPS-binding protein in IBD	39
CD38+HLA-DR+ CD8 T cells	T cell	CD14–CD3+CD8+CD38+HLA-DR+	Highly activated phenotype; abundance correlated with levels of LPS-binding protein in IBD	39
CD43+ T cells	T cell	CD14–CD3+CD43+	CD43 expression enhances T cell activation and inhibits apoptosis	40,41
Central memory CD4 T cells	T cell	CD14–CD3+CD4+CD27+CD45RO+		42
CXCR5–PD-1– CD45RO+ CD4 T cells	T cell	CD14–CD3+CD4+CD45RO+CXCR5–PD-1–	Produce more IL-2 and less IL-21 and CXCL13 compared to Tph cells	43
Effector CD4 T cells	T cell	CD14–CD3+CD4+CD27–CD45RO–	Expanded in IBD	42
Effector CD8 T cells	T cell	CD14–CD3+CD8+CD27–CD45RO–	Expanded in IBD	42
Effector memory CD4 T cells	T cell	CD14–CD3+CD4+CD27–CD45RO–	Expanded in IBD	42
Effector memory CD8 T cells	T cell	CD14–CD3+CD8+CD27–CD45RO+	Expanded in IBD	42
Naïve CD4 T cells	T cell	CD14–CD3+CD4+CD27+CD45RO–		42
T peripheral helper (Tph) cells	T cell	CD14–CD3+CD4+CD45RO+CXCR5–PD-1+	Provide help to pathogenic autoreactive B cells; expanded in RA	43

Supplemental Table 6. Cell subsets presented in the study. Key gating features indicate gating strategy used to identify cells of interest from gated non-basophils; see Supplemental Figures 1 and 2 for further gating scheme.

SUPPLEMENTAL INFORMATION REFERENCES

1. Baumgarth, N. The double life of a B-1 cell: self-reactivity selects for protective effector functions. *Nat Rev Immunol* **11**, 34-46 (2011).
2. Kessel, A., *et al.* Human CD19(+)CD25(high) B regulatory cells suppress proliferation of CD4(+) T cells and enhance Foxp3 and CTLA-4 expression in T-regulatory cells. *Autoimmun Rev* **11**, 670-677 (2012).
3. Mizoguchi, A. & Bhan, A.K. Immunobiology of B Cells in Inflammatory Bowel Disease. in *Crohn's Disease and Ulcerative Colitis: From Epidemiology and Immunobiology to a Rational Diagnostic and Therapeutic Approach* (ed. Baumgart, D.C.) 161-168 (Springer-Verlag, New York, 2012).
4. Vadasz, Z., Haj, T., Kessel, A. & Toubi, E. B-regulatory cells in autoimmunity and immune mediated inflammation. *FEBS Lett* **587**, 2074-2078 (2013).
5. Bing, X., Linlang, L. & Keyan, C. Decreased Breg/Th17 Ratio Improved the Prognosis of Patients with Ulcerative Colitis. *Can J Gastroenterol Hepatol* **2018**, 5760849 (2018).
6. Sanz, I., Wei, C., Lee, F.E. & Anolik, J. Phenotypic and functional heterogeneity of human memory B cells. *Semin Immunol* **20**, 67-82 (2008).
7. Yacyshyn, B.R. & Pilarski, L.M. Expression of CD45RO on circulating CD19+ B-cells in Crohn's disease. *Gut* **34**, 1698-1704 (1993).
8. Muroi, K., *et al.* CD56 expression in B-cell lymphoma. *Leuk Res* **22**, 201-202 (1998).
9. Henneken, M., Dorner, T., Burmester, G.R. & Berek, C. Differential expression of chemokine receptors on peripheral blood B cells from patients with rheumatoid arthritis and systemic lupus erythematosus. *Arthritis Res Ther* **7**, R1001-1013 (2005).
10. Stokkers, P.C., Reitsma, P.H., Tytgat, G.N. & van Deventer, S.J. HLA-DR and -DQ phenotypes in inflammatory bowel disease: a meta-analysis. *Gut* **45**, 395-401 (1999).
11. Wu, Y.C., Kipling, D. & Dunn-Walters, D.K. The relationship between CD27 negative and positive B cell populations in human peripheral blood. *Front Immunol* **2**, 81 (2011).
12. Fecteau, J.F., Cote, G. & Neron, S. A new memory CD27-IgG+ B cell population in peripheral blood expressing VH genes with low frequency of somatic mutation. *J Immunol* **177**, 3728-3736 (2006).
13. Fleischer, S.J., *et al.* Increased frequency of a unique spleen tyrosine kinase bright memory B cell population in systemic lupus erythematosus. *Arthritis Rheumatol* **66**, 3424-3435 (2014).
14. Wei, C., *et al.* A new population of cells lacking expression of CD27 represents a notable component of the B cell memory compartment in systemic lupus erythematosus. *J Immunol* **178**, 6624-6633 (2007).
15. Moir, S., *et al.* Evidence for HIV-associated B cell exhaustion in a dysfunctional memory B cell compartment in HIV-infected viremic individuals. *J Exp Med* **205**, 1797-1805 (2008).
16. Rojas, O.L., Narvaez, C.F., Greenberg, H.B., Angel, J. & Franco, M.A. Characterization of rotavirus specific B cells and their relation with serological memory. *Virology* **380**, 234-242 (2008).
17. Thibult, M.L., *et al.* PD-1 is a novel regulator of human B-cell activation. *Int Immunol* **25**, 129-137 (2013).
18. Kaminski, D.A., Wei, C., Qian, Y., Rosenberg, A.F. & Sanz, I. Advances in human B cell phenotypic profiling. *Front Immunol* **3**, 302 (2012).
19. Collin, M., McGovern, N. & Haniffa, M. Human dendritic cell subsets. *Immunology* **140**, 22-30 (2013).
20. Baban, B., *et al.* A minor population of splenic dendritic cells expressing CD19 mediates IDO-dependent T cell suppression via type I IFN signaling following B7 ligation. *Int Immunol* **17**, 909-919 (2005).
21. Beyer, M., *et al.* Sustained increases in numbers of pulmonary dendritic cells after respiratory syncytial virus infection. *J Allergy Clin Immunol* **113**, 127-133 (2004).
22. Villadangos, J.A., *et al.* MHC class II expression is regulated in dendritic cells independently of invariant chain degradation. *Immunity* **14**, 739-749 (2001).
23. Schefold, J.C., *et al.* Diminished HLA-DR expression on monocyte and dendritic cell subsets indicating impairment of cellular immunity in pre-term neonates: a prospective observational analysis. *J Perinat Med* **43**, 609-618 (2015).
24. Aranami, T., Miyake, S. & Yamamura, T. Differential expression of CD11c by peripheral blood NK cells reflects temporal activity of multiple sclerosis. *J Immunol* **177**, 5659-5667 (2006).
25. Romee, R., *et al.* NK cell CD16 surface expression and function is regulated by a disintegrin and metalloprotease-17 (ADAM17). *Blood* **121**, 3599-3608 (2013).
26. Holtan, S.G., Creedon, D.J., Thompson, M.A., Nevala, W.K. & Markovic, S.N. Expansion of CD16-negative natural killer cells in the peripheral blood of patients with metastatic melanoma. *Clin Dev Immunol* **2011**, 316314 (2011).
27. Huang, Y. & Chen, Z. Inflammatory bowel disease related innate immunity and adaptive immunity. *Am J Transl Res* **8**, 2490-2497 (2016).
28. Mallone, R., *et al.* Signaling through CD38 induces NK cell activation. *Int Immunol* **13**, 397-409 (2001).
29. Sconocchia, G., *et al.* CD38 triggers cytotoxic responses in activated human natural killer cells. *Blood* **94**, 3864-3871 (1999).
30. Rah, S.Y., Kwak, J.Y., Chung, Y.J. & Kim, U.H. ADP-ribose/TRPM2-mediated Ca2+ signaling is essential for cytolytic degranulation and antitumor activity of natural killer cells. *Sci Rep* **5**, 9482 (2015).
31. Aguado, E., Santamaria, M., Gallego, M.D., Pena, J. & Molina, I.J. Functional expression of CD43 on human natural killer cells. *J Leukoc Biol* **66**, 923-929 (1999).
32. Nieto, M., *et al.* Signaling through CD43 induces natural killer cell activation, chemokine release, and PYK-2 activation. *Blood* **94**, 2767-2777 (1999).
33. Montaldo, E., *et al.* Human NK cell receptors/markers: a tool to analyze NK cell development, subsets and function. *Cytometry A* **83**, 702-713 (2013).
34. Liu, Y., *et al.* Increased expression of programmed cell death protein 1 on NK cells inhibits NK-cell-mediated anti-tumor function and indicates poor prognosis in digestive cancers. *Oncogene* **36**, 6143-6153 (2017).
35. Chen, Y.G., *et al.* CD38 is required for the peripheral survival of immunotolerogenic CD4+ invariant NK T cells in nonobese diabetic mice. *J Immunol* **177**, 2939-2947 (2006).
36. Farr, A.R., Wu, W., Choi, B., Cavalcoli, J.D. & Laouar, Y. CD1d-unrestricted NKT cells are endowed with a hybrid function far superior than that of iNKT cells. *Proc Natl Acad Sci U S A* **111**, 12841-12846 (2014).
37. Monticelli, L.A., *et al.* Transcriptional regulator Id2 controls survival of hepatic NKT cells. *Proc Natl Acad Sci U S A* **106**, 19461-19466 (2009).
38. Ladd, M., *et al.* Natural killer T cells constitutively expressing the interleukin-2 receptor alpha chain early in life are primed to respond to lower antigenic stimulation. *Immunology* **131**, 289-299 (2010).
39. Funderburg, N.T., *et al.* Circulating CD4(+) and CD8(+) T cells are activated in inflammatory bowel disease and are associated with plasma markers of inflammation. *Immunology* **140**, 87-97 (2013).
40. He, Y.W. & Bevan, M.J. High level expression of CD43 inhibits T cell receptor/CD3-mediated apoptosis. *J Exp Med* **190**, 1903-1908 (1999).
41. Park, J.K., *et al.* Enhancement of T-cell activation by the CD43 molecule whose expression is defective in Wiskott-Aldrich syndrome. *Nature* **350**, 706-709 (1991).
42. Sallusto, F., Geginat, J. & Lanzavecchia, A. Central memory and effector memory T cell subsets: function, generation, and maintenance. *Annu Rev Immunol* **22**, 745-763 (2004).
43. Rao, D.A., *et al.* Pathologically expanded peripheral T helper cell subset drives B cells in rheumatoid arthritis. *Nature* **542**, 110-114 (2017).
