# Supplementary material for: Mass cytometry reveals systemic and local immune signatures that distinguish inflammatory bowel diseases
Source: Nat Commun. 2019 Jun 19;10:2686. doi: 10.1038/s41467-019-10387-7 (PMC6584653; doi:10.1038/s41467-019-10387-7)
Supplement: Supplementary file 2 — Reporting Summary [file 41467_2019_10387_MOESM2_ESM.pdf]

nature research

Corresponding author(s): Aida Habtezion

Last updated by author(s): Apr 20, 2019

Reporting Summary

Nature Research wishes to improve the reproducibility of the work that we publish. This form provides structure for consistency and transparency in reporting. For further information on Nature Research policies, see Authors & Referees and the Editorial Policy Checklist.

Statistics

For all statistical analyses, confirm that the following items are present in the figure legend, table legend, main text, or Methods section.

n/a	Confirmed	
☐	☒	The exact sample size (*n*) for each experimental group/condition, given as a discrete number and unit of measurement
☐	☒	A statement on whether measurements were taken from distinct samples or whether the same sample was measured repeatedly
☐	☒	The statistical test(s) used AND whether they are one- or two-sided<br>*Only common tests should be described solely by name; describe more complex techniques in the Methods section.*
☐	☒	A description of all covariates tested
☐	☒	A description of any assumptions or corrections, such as tests of normality and adjustment for multiple comparisons
☐	☒	A full description of the statistical parameters including central tendency (e.g. means) or other basic estimates (e.g. regression coefficient) AND variation (e.g. standard deviation) or associated estimates of uncertainty (e.g. confidence intervals)
☐	☒	For null hypothesis testing, the test statistic (e.g. F, t, r) with confidence intervals, effect sizes, degrees of freedom and P value noted<br>*Give P values as exact values whenever suitable.*
☒	☐	For Bayesian analysis, information on the choice of priors and Markov chain Monte Carlo settings
☒	☐	For hierarchical and complex designs, identification of the appropriate level for tests and full reporting of outcomes
☐	☒	Estimates of effect sizes (e.g. Cohen's *d*, Pearson's *r*), indicating how they were calculated

Our web collection on statistics for biologists contains articles on many of the points above.

Software and code

Policy information about availability of computer code

Data collection	For CyTOF, bead normalized sample files were obtained from the Helios instrument using on-board software. For flow cytometry, raw files were obtained from the BD LSR II instrument.
Data analysis	FlowJo (Version 10) was used for cleaning up files, concatenating files, and calculating manual gates and statistics. VorteX (Version 29-Jun-2017-rev2) was used to find the optimal cluster number for use in subsequent analyses. Stanford Cytobank was used to perform viSNE, CITRUS and Spade analyses. GraphPad PRISM (Version 7) was used to plot some figures and conduct some targeted statistical tests, and Microsoft Excel 2016 was used for some basic data manipulation. Morpheus (https://software.broadinstitute.org/morpheus) was utilized for constructing heatmaps and Pearson correlation maps based on significant parameters selected using T-tests. RStudio (Version 1.1.447 with R Version 3.3.2) was used for further statistical analysis. When correcting for multiple testing, the p.adjust package with BH FDR correction was used in R. Correlation tests were based on Pearson correlations using cor.test in R. For receiver operating characteristic (ROC) analysis, generalized linear models (GLM) were constructed using glm in R. For each ROC curve, the area under the curve and 95% confidence intervals were calculated using package ROCR in R. Optimal cutoff values and associated sensitivity and specificity values for GLMs were calculated using the OptimalCutpoints package in R with the Youden index method. Analysis of covariance (ANCOVA) was conduced using the aov package in R.

For manuscripts utilizing custom algorithms or software that are central to the research but not yet described in published literature, software must be made available to editors/reviewers. We strongly encourage code deposition in a community repository (e.g. GitHub). See the Nature Research guidelines for submitting code & software for further information.

Data

Policy information about availability of data

All manuscripts must include a data availability statement. This statement should provide the following information, where applicable:

- Accession codes, unique identifiers, or web links for publicly available datasets
- A list of figures that have associated raw data
- A description of any restrictions on data availability

The datasets generated during the current study are available from the corresponding author on reasonable request.

Field-specific reporting

Please select the one below that is the best fit for your research. If you are not sure, read the appropriate sections before making your selection.

☒ Life sciences ☐ Behavioural & social sciences ☐ Ecological, evolutionary & environmental sciences

For a reference copy of the document with all sections, see nature.com/documents/nr-reporting-summary-flat.pdf

Life sciences study design

All studies must disclose on these points even when the disclosure is negative.

Sample size	Sample sizes were maximized based on availability of clinical samples. No prospective sample size calculations were performed.
Data exclusions	Samples were only excluded from statistical analyses based on pre-established exclusion criteria, such as if the sample did not meet the clinical criteria of the comparison in question, or if the sample was from a patient treated with an alpha4beta7 antagonist for comparisons involving trafficking receptors.
Replication	In general, statistical analyses were conducted for groups of samples meeting specific criteria but not for replicates. Where indicated, findings from cohort 1 blood samples were validated using cohort 2 samples, although they were not always reproduced (as discussed in the text), likely due to the smaller sample size of cohort 2 and that all patients in cohort 2 were in clinical remission.
Randomization	Samples were randomly allocated into CyTOF batches. The clinical data associated with each patient sample determined the disease comparison groups used in analyses based on features such as disease diagnosis, disease behavior, disease location, etc.
Blinding	CyTOF operators were blinded to the sample groups at the time of data acquisition. Blinding was not possible during analysis since comparisons based on clinical features were central to the study.

Reporting for specific materials, systems and methods

We require information from authors about some types of materials, experimental systems and methods used in many studies. Here, indicate whether each material, system or method listed is relevant to your study. If you are not sure if a list item applies to your research, read the appropriate section before selecting a response.

Materials & experimental systems

n/a	Involved in the study	
☐	☒	Antibodies
☒	☐	Eukaryotic cell lines
☒	☐	Palaeontology
☒	☐	Animals and other organisms
☐	☒	Human research participants
☒	☐	Clinical data

Methods

n/a	Involved in the study	
☒	☐	ChIP-seq
☐	☒	Flow cytometry
☒	☐	MRI-based neuroimaging

Antibodies

Antibodies used	The following anti-human antibodies were used for CyTOF: 141Pr-CD20 (clone 2H7, BioLegend 302343 antibody and Fluidigm 201141A metal conjugation kit), 142Nd-CD19 (clone HIB19, Fluidigm 3142001B), 143Nd-CD5 (clone UCHT2, Fluidigm 3143007B), 144Nd-pPLCgamma2[pY759] (clone K86-689.37, Fluidigm 3144015A), 145Nd-CD4 (clone RPA-T4, Fluidigm 3145001B), 146Nd-IgD (clone IA6-2, Fluidigm 3146005B), 147Nd-pSTAT5[Y694] (clone 47, Fluidigm 3150005A), 148Nd-IgA (polyclonal, Fluidigm 3148007B), 149Sm-CD25 (clone 2A3, Fluidigm 3149010B), 150Nd-CD43 (clone 84-3C1, Fluidigm 3150006B), 151Eu-CD123 (clone 6H6, Fluidigm 3151001B), 152Sm-Akt[S473] (clone D9E, Fluidigm 3152005A), 153Eu-pSTAT1[Y701] (clone 4a, Fluidigm 3153005A), 154Sm-GPR15-PE (clone 373004, BioLegend custom antibody and Fluidigm 201154A metal conjugation kit), 155Gd-CD27 (clone L128, Fluidigm 3155001B), 156Gd-p-p38[T180/Y182] (clone D3F9, Fluidigm 3156002A), 157Gd-CD24 (clone ML-5, BioLegend 311127 antibody and Stanford University Human Immune Monitoring Center custom metal conjugation), 158Gd-

pSTAT3[Y705] (clone 4, Fluidigm 3158005A), 159Tb-pMAPKAPK2 (clone 27B7, Fluidigm 3159010A), 160Gd-CD14 (clone M5E2, Fluidigm 3160001B), 161Dy-CCR9 (clone L053E8, BioLegend 358902 antibody and Fluidigm 201161A metal conjugation kit), 162Dy-CD11c (clone Bu15, Fluidigm 3162005B), 163Dy-CD56 (clone NCAM16.2, Fluidigm 3163007B), 164Dy-IkappaBa (clone L35A5, Fluidigm 3164004A), 165Ho-pCREB[S133] (clone 87G3, Fluidigm 3165009A), 166Er-CD16 (clone B73.1, Stanford University Human Immune Monitoring Center custom antibody conjugate), 167Er-CD38 (clone HIT2, Fluidigm 3167001B), 168Er-CD8alpha (clone SK1, Fluidigm 3168002B), 169Tm-CCR1 (clone 5F10B29, BioLegend 362902 antibody and Fluidigm 201169A metal conjugation kit), 170Er-CD3 (clone UCHT1, Fluidigm 3170001B), 171Yb-pERK1/2[T202/Y204] (clone D13.14.4E, Fluidigm 3171010A), 172Yb-CD45RO (clone UCHL1, BioLegend 304239 antibody and Fluidigm 201172A metal conjugation kit), 173Yb-alpha4beta7 (clone Act1, NIH AIDS Reagent Program 11718 antibody and Fluidigm 201173A metal conjugation kit), 174Yb-HLA-DR (clone L243, Fluidigm 3174001B), 175Lu-pS6 (clone N7548, Fluidigm 3175009A), 176Yb-CD127 (clone A019D5, Fluidigm 3176004B), 209Bi-CD11b (clone ICRF44, Fluidigm 3209003B), 89Y-CD45 (clone HI30, Fluidigm 3089003B), 143Nd-HLA-DR (clone L243, Fluidigm 3143013B), 152Sm-TCRgammadelta (clone 11F2, Fluidigm 3152008B), 171Yb-CXCR5 (clone RF8B2, Fluidigm 3171014B), 174-CD94 (clone HP-3D9, Fluidigm 3174015B), 175Yb-PD-1 (clone EH12.2H7, Fluidigm 3175008B).
The following antibodies were used for flow cytometry: CD123-BV421 (clone 6H6, BioLegend 306018), CD14-BV510 (clone M5E2, BioLegend 301842), CD11c-PerCP-Cy5.5 (clone Bu15, BioLegend 337210), HLA-DR-BV605 (clone L243, BioLegend 307640), CD19-PE (clone HIB19, BioLegend 302208), CD3-APC (clone UCHT1, BioLegend 300412), CD20-BV711 (clone 2H7, BioLegend 302342).

Validation

All antibodies were used according to the manufacturer's instructions and validated using healthy donor human PBMCs.

Human research participants

Policy information about studies involving human research participants

Population characteristics

Subjects age 18 to 75 with an IBD-specializing gastroenterologist-confirmed diagnosis of inflammatory bowel disease (except healthy controls) were recruited, excluding those who were pregnant, had other autoimmune or inflammatory diseases (except for extra-intestinal manifestations of IBD), had a malignancy, had an active infection at the time of enrollment, had undergone surgery within one month of enrollment, had a blood transfusion within one month of enrollment, had received an organ or bone marrow transplant, or were unable to provide informed consent. All clinical data for subjects was current at the time of sample collection. Flare and remission classifications were defined by gastroenterologist assessment for standard of care.

Recruitment

Subjects were recruited by investigators certified to conduct clinical research at the Stanford University Medical Center. All subjects were in-patients or out-patients of Stanford Medicine. Potential selection bias could relate to times of day and days of the week preferred for sample collection due to logistical requirements.

Ethics oversight

All blood and tissue samples were collected under provision of the Stanford Institutional Review Board.

Note that full information on the approval of the study protocol must also be provided in the manuscript.

Flow Cytometry

Plots

Confirm that:

☒ The axis labels state the marker and fluorochrome used (e.g. CD4-FITC).

☒ The axis scales are clearly visible. Include numbers along axes only for bottom left plot of group (a 'group' is an analysis of identical markers).

☒ All plots are contour plots with outliers or pseudocolor plots.

☒ A numerical value for number of cells or percentage (with statistics) is provided.

Methodology

Sample preparation

Sample collection: Blood samples were collected by standard of care venipuncture; three vacutainers with sodium heparin (BD cat. #366480) were filled with blood and kept at room temperature until processing, which occurred within two hours. Tissue samples were collected during standard of care endoscopic procedures (within two hours of paired blood sample collection) using biopsy forceps rinsed in sterile saline to remove any residual formalin. Two to four tissue bites per site were collected and deposited in 3mL of sterile PBS without calcium or magnesium in a 5mL Eppendorf tube and kept at room temperature if processed within 30 minutes or kept on ice if processed within 2 hours. Biopsies were excluded if they were sampled from sites exposed to methylene blue during endoscopic procedures. Samples from inflamed and uninflamed tissues were initially identified by the endoscopist and subsequently confirmed by a blinded pathologist.

Blood leukocyte isolation and cryopreservation: Blood was centrifuged in vacutainers used for collection at 2000 RPM for 10 minutes. Plasma was aspirated from the top and frozen at –80ºC in 1mL aliquots in cryovials (Thermo Fisher Scientific cat. #375418) using a freeze controller (Bel-Art Products cat. #F18844-0000) pre-chilled to –4ºC according to the manufacturer's instructions. The remaining blood was diluted 1:1 in PBS without calcium or magnesium, layered over 15mL of Ficoll-Paque (GE Healthcare cat. #17-1440-03) in an Accuspin tube (Sigma-Aldrich cat. #A2055), and centrifuged at 2000 RPM for 20 minutes at 21ºC with acceleration at five and break at zero. The buffy coat leukocyte layer was collected and washed twice in 50mL PBS without calcium or magnesium by centrifuging at 2000 RPM for 10 minutes. Cells were counted, washed again, and resuspended in Recovery Cell Culture Freezing Medium (Thermo Fisher Scientific cat. #12648010) at 3.5–10?106 cells/mL in 1mL aliquots, transferred to a freeze controller (Bel-Art Products cat. #F18844-0000) pre-chilled to –4ºC according to the manufacturer's instructions, stored at –80ºC for one to seven days, and then transferred to liquid nitrogen for storage.

Tissue leukocyte isolation and cryopreservation: Combined biopsy bite tissue samples from the same site were washed in HBSS without calcium or magnesium supplemented with 2% BSA and then transferred to 5mL of RPMI with HEPES, 5% BSA, collagenase IV at 0.7mg/mL (Sigma-Aldrich cat. #C5138), and DNase I at 50µg/mL (Worhington Biochemical cat. #LS002060) at 37ºC on a magnetic stirrer at 400 RPM for 40 minutes in a small glass jar with a magnetic stirrer. The cell suspension was strained through a 100µm filter (Falcon cat. #352360), quenched with 5mL of RPMI with HEPES and 5% BSA, centrifuged at 560g for 10 minutes, and kept on ice. The remaining undigested tissue was again resuspended in 5mL of RPMI with HEPES, 5% BSA, collagenase IV at 0.7mg/mL (Sigma-Aldrich cat. #C5138), and DNase I at 50µg/mL (Worhington Biochemical cat. #LS002060) at 37ºC on a magnetic stirrer at 400 RPM for 40 minutes in a small glass jar with a magnetic stirrer. The material was again strained through a 100µm filter (Falcon cat. #352360), quenched with 5mL of RPMI with HEPES and 5% BSA, and centrifuged at 560g for 10 minutes. The combined cell suspensions were then resuspended in 8mL of 40% Percoll, which was made by preparing a mixture of 10% 10X PBS and 90% Percoll (GE Healthcare cat. #17-0891-01) and then diluting this in RPMI with HEPES and 5% BSA. The 40% Percoll cell suspension was overlaid on 2mL of 80% Percoll (prepared in a manner analogous to that previously described for 40%), centrifuged at 560g for 20 minutes with acceleration of four and break of one at room temperature. The buffy coat leukocyte layer was collected and washed in 15mL of RPMI with HEPES and 5% BSA. Cells were counted, washed again, resuspended in 500µL of Recovery Cell Culture Freezing Medium (Thermo Fisher Scientific cat. #12648010) per tissue sample site, transferred to a freeze controller (Bel-Art Products cat. #F18844-0000) pre-chilled to –4ºC according to the manufacturer's instructions, stored at –80ºC for one to seven days, and then transferred to liquid nitrogen for storage.

Mass cytometry: Phospho CyTOF was conducted at the Stanford Human Immune Monitoring Center using viably cryopreserved leukocyte samples according to published methods54, unless otherwise noted, in batches of 10–20 samples per day on the same Helios instrument using the same operator. All antibody conjugates were validated for accurate detection of their respective antigens and to ensure minimal isotope spillover by us, the Stanford Human Immune Monitoring Center, and/or in the literature using flow cytometry with antibody clones and mass cytometry with antibody-metal conjugates (Supplemental Table 6). Beads (Fluidigm cat. #201078) were spiked into each sample for subsequent normalization using the Helios instrument software, and no cell stimulation or barcoding were used.
In brief, cells were thawed, washed twice in 10mL of complete RPMI with 1:10,000 benzonase (Pierce Antibodies cat. #88701), and washed again in complete RPMI. Cells were counted and 1?106 live cells were used for staining; for tissue samples with less than 1?106 live cells recovered, thawed mouse splenocytes (processed as described above for human blood) were spiked in to reach 1?106 live cells per sample. Cells were transferred to deep well plates, washed in RPMI and then incubated for 3 minutes at room temperature in 100µL of 1:5000 cisplatin live/dead stain (Fluidigm cat. #201064) in RPMI. Cells were washed twice with complete RPMI, resuspended in 200µL complete RPMI, and rested for 1 hour at 37ºC in a CO_2 incubator. For surface staining, cells were washed with CyFACS buffer, stained with anti-?4?7 antibody in 25µL total volume per sample for 20 minutes at room temperature, washed twice with CyFACS, and fixed with 200µL of 2% PFA in PBS for 10 minutes at room temperature. Cells were washed twice with 1mL of CyFACS per well and centrifuged at 2000 RPM for 8 minutes at 4ºC. Samples were stained with surface antibody cocktails in a total staining volume of 20µL per sample for 30 minutes at room temperature, washed twice with 1mL of CyFACS buffer per well, and centrifuged at 974g for 8 minutes at 4ºC. Cells were fixed again in 100µL of 4% PFA in PBS for 10 minutes at room temperature, washed with PBS, permeabilized with 600µL of –20ºC methanol per sample, and stored overnight at –80ºC. The next day, samples were resuspended in 1mL CyFACS buffer and centrifuged at 974g for 10 minutes at 4ºC, and then washed again in PBS. Samples were stained with intracellular antibody cocktails in a total staining volume of 20µL per sample for 30 minutes at room temperature, washed in 1mL of CyPBS, and 300µL of Ir-intercalator (Fluidigm cat. #201192B) diluted according to the manufacturer's instructions was added to each sample for 20 minutes at room temperature. Samples were washed once with PBS, twice with water, spiked with beads according to the manufacturer's instructions, and then analyzed on a Helios instrument. Approximately 100,000 or all possible events (whichever lower) were acquired for each sample.

Flow cytometry: Flow cytometry was conducted at the Stanford Shared FACS Facility (SSFF) on a BD LSRII instrument in accordance with standard methods. In brief, cells were thawed, washed once in complete RPMI, incubated in 1mL complete RPMI with 2.5mM $MgCl_2$ (ThermoFisher cat. #AM9530G) and 0.5mg/mL DNase I (Worthington Biochemical cat. #LS002060) for 10 minutes at room temperature, and washed in 10mL of complete RPMI. Cells were counted and 1?106 or 2?106 live cells were aliquoted for staining. Cells were washed with FACS buffer (HBSS without calcium or magnesium and supplemented with 2% BSA). Cells were resuspended in 100µL of 1:500 Zombie Green fixable viability stain (BioLegend cat. #423111) in PBS without calcium or magnesium and incubated for 15 minutes at room temperature in the dark. Cells were washed with FACS buffer, resuspended in a master mix of fluorochrome-conjugated antibodies (Supplemental Fig. 4B) using the supplier-recommended 5µL of each antibody per 1?106 cells, and incubated for 30 minutes at 4C in the dark. Cells were washed with FACS buffer, resuspended in 100µL of FACS buffer, and kept on ice in the dark until sample analysis. Approximately 1?106 or all possible events (whichever lower) were acquired for each sample. For single color compensation controls, one drop of negative control and one drop of anti-mouse compensation beads (BD cat. #552843) were incubated in 100µL of FACS buffer and 5µL of antibody for 15 minutes at room temperature in the dark and then kept on ice in the dark until analysis.

Instrument

All CyTOF data was collected on a Fluidigm Helios instrument. All flow cytometry data was collected on a BD LSRII instrument.

Software

All CyTOF data was collected using Helios software on the instrument. All flow cytometry data was collected using BD FACS DIVA software. Please see "Data analysis" section above for a description of how the data was analyzed.

Cell population abundance

No cell populations were sorted.

Gating strategy

Mass cytometry: First, non-bead events were gated as bead-negative and DNA-positive. Then, intact cells were gated as DNA- and DNA-1 -high, live singlets were gated as Cisplatin-negative and Event_length-low, and if mouse cells were spiked into the sample then human cells were gated as human CD45-positive. Next, basophils, non-basophils, lymphocytes, monocytes, and numerous subsets therof were gated according to the gating schemes in Supplemental Figures 1 and 2.

Flow cytometry: First, lymphocytes were gating using the SSC-A vs. FSC-A plot. Then, single cells were gated using the FSC-H vs. FSC-A plot, and live cells were gated as Zombie Green negative. Then, basophils, non-basophils, and other cells were gated as described above for mass cytometry data.

☒ Tick this box to confirm that a figure exemplifying the gating strategy is provided in the Supplementary Information.
